# Formulation of Chitosan-Coated Apigenin Bilosomes: In Vitro Characterization, Antimicrobial and Cytotoxicity Assessment

**DOI:** 10.3390/polym14050921

**Published:** 2022-02-25

**Authors:** Syed Sarim Imam, Sultan Alshehri, Mohammad A. Altamimi, Raed Khalid Hassan Almalki, Afzal Hussain, Sarah I. Bukhari, Wael A. Mahdi, Wajhul Qamar

**Affiliations:** 1 Department of Pharmaceutics, College of Pharmacy, King Saud University, Riyadh 11451, Saudi Arabia; simam@ksu.edu.sa (S.S.I.); maltamimi@ksu.edu.sa (M.A.A.); 439106309@student.ksu.edu.sa (R.K.H.A.); amohammed2@ksu.edu.sa (A.H.); sbukhari@ksu.edu.sa (S.I.B.); wmahdi@ksu.edu.sa (W.A.M.); 2 Department of Pharmacology and Toxicology, College of Pharmacy, King Saud University, Riyadh 11451, Saudi Arabia; wqidris@ksu.edu.sa

**Keywords:** apigenin, antimicrobial, bilosomes, cell viability, chitosan

## Abstract

We prepared apigenin (APG)-loaded bilosomes (BLs) and evaluated them for vesicle size, zeta-potential and encapsulation efficiency. The formulations were prepared with cholesterol (CHL), sodium deoxy cholate (SDC), Tween 80 (T80) and phosphatidylcholine (PC) using solvent evaporation method. The prepared formulations showed the optimum result was coated with much mucoadhesive polymer chitosan (CH, 0.25 and 0.5% *w*/*v*). The chitosan-coated bilosomes (CH-BLs) were further evaluated for surface morphology, drug–polymer interaction, mucoadhesion, permeation, antimicrobial activity and cell viability. The prepared APG-BLs showed nano-metric size (211 ± 2.87 nm to 433 ± 1.98 nm), polydispersibility index <0.5, negative zeta potential (−15 to −29 mV) and enhanced encapsulation efficiency (69.5 ± 0.93 to 81.9 ± 1.3%). Based on these findings, selected formulation (F2) was further coated with chitosan and showed a marked increase in vesicle size (298 ± 3.56 nm), a positive zeta potential (+17 mV), superior encapsulation efficiency (88.1 ± 1.48%) and improved drug release (69.37 ± 1.34%). Formulation F2C1 showed significantly enhanced permeation and mucoadhesion (*p* < 0.05) compared to formulation F2 due to the presence of CH as a mucoadhesive polymer. The presence of CH on the surfaces of BLs helps to open the tight membrane junctions and leads to enhanced permeation. A TEM study revealed non-aggregated smooth surface vesicles. The antimicrobial and cell viability assessment revealed better effects in terms of zone of inhibition and cell line assessment against two different cancer cell line. From the study, it can be concluded that APG-CHBLs could be a superior alternative to conventional delivery systems.

## 1. Introduction

Apigenin is a flavonoid obtained from fruits, vegetables, and sprouts. The therapeutic efficacy of the APG is restricted due to its low water solubility, quick metabolism and low bioavailability. APG has numerous pharmacological activities. It is non-mutagenic and can exert anti-inflammatory, anti-viral [1] and anti-carcinogenic effects [2,3]. One study reported that APG suppresses the proliferation and migration and induces the apoptosis of breast cancer cells [3]. Various APG-loaded nano-delivery systems have been reported in the literature. APG-loaded phytosomes have been prepared and optimized using full factorial design [2]. They had enhanced solubility, dissolution and in vivo activity. The solubility was enhanced 36-fold, and a higher release rate was achieved. The formulation was also evaluated for bioavailability, and effects shown a significant enhancement. A liver function test was also performed, and a significant enhancement in the profile was achieved after encapsulation into phytosomes. In another study, APG-loaded nanoparticles were prepared via anti-solvent precipitation method to enhance solubility and bioavailability [4]. The prepared APG-NPs showed nano-metric size (159.2 nm) and enhanced solubility and dissolution. The biological activity results revealed an about 5-fold increase in oral bioavailability and no toxic effects (lower IC_50_ value than pure APG). Shen et al. prepared APG-loaded ethosomes and further optimized them using a design of experiments method for topical delivery [5]. The prepared ethosomes were evaluated with tweaks to various parameters, and the results revealed enhanced encapsulation efficiency, skin deposition and transdermal flux. They also found a strong effect on cyclooxygenase 2 levels in the skin of inflamed mice. Another research group prepared APG-loaded liposomes via lipid film hydration and evaluated their effect in colorectal cancer [6]. The study results revealed potential clinical therapeutic efficacy. For the colorectal cancer cell line, apigenin induced a G2/M arrest. It also had an effect on tumor growth: liposomal apigenin had a greater tumor inhibitory effect.

The effect of flavonoids can be enhanced by encapsulating them in a nano-sized delivery system. In recent years, numerous studies have described use of lipid-based nanoformulations to enhance solubility and dissolution. These delivery systems have advantage of a higher effective surface area for drug absorption. Various lipid-based nano-vesicles formulations, such as liposome, niosome, ethosomes, transferosomes and bilosomes [7], have been reported to provide enhanced therapeutic efficacy to poorly water-soluble drugs via different routes. Recently, concept of bilosomes (BLs) was widely accepted due to their superior intestinal stability and deformability, which helps to promote drug absorption in intestinal region [8]. They are modified niosomes containing bile salts in lipid bilayer, and they show greater stability. It is composed of cholesterol, surfactant, lipids and bile salts (sodium glycocholate, sodium deoxycholate). These bile salts have been reported to enhance intestinal permeability and gastrointestinal stability to several drugs, including acyclovir [7] and carvedilol [9]. The use of cholesterol enables their membrane permeability, adds rigidity to vesicle walls and also promotes higher encapsulation.

In recent times, chitosan (CH) has been widely used as a natural cationic polymer due to its bioadhesive, biocompatible and biodegradable nature. CH has been reported for enhanced permeation through intestinal mucosal barrier [10]. The cationic charge of CH can interact with a negatively charged cell membrane to open tight epithelial junctions [11]. CH, with its cationic charge, can easily attach to anionic charged macromolecules, which form a protective layer around it and also increase the cellular permeability [12,13]. It is also reported to have anti-bacterial activity by acting on cell walls of bacteria [14,15]. It acts by enhancing blood clotting (hemostatic) [16]. The NH_3_^+^ group in chitosan causes it to be adsorbed on microorganisms’ cell walls via electrostatic interaction. This leads to cell wall damage and leakage of macromolecules from bacteria [14,17].

**Figure 1:**
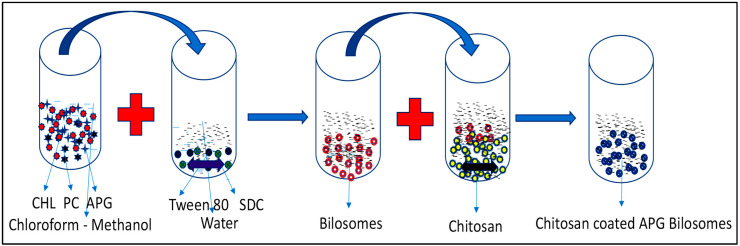
Schematic representation of the formulation of chitosan-coated apigenin bilosomes.

In this research, we prepared apigenin bilosomes (APG-BLs) via solvent evaporation method (Figure 1). The selected APG-BLs showed low vesicle size, high encapsulation efficiency and excellent drug release. We then coated them with the cationic polymer chitosan. The surface-modified bilosomes (APG-CHBLs) were also evaluated.

## 2. Materials and Methods

### Materials

Beijing Mesochem Technology Co. Pvt. Ltd. (Beijing, China) and Spectrochem Pvt. Ltd. (Mumbai, India) supplied apigenin and chitosan, respectively. The surfactants cholesterol (CHL), tween 80 (T80), sodium deoxy cholate (SDC), phosphatidyl choline (PC) and chitosan (CH) were purchased from Alpha Chemika (Mumbai, India) and UFC Biotech (New York, NY 14228, USA). The lung cancer cell line (A549) and breast cancer cell line (MCF7) were obtained from the German Collection of Microorganisms and Cell Cultures (DSMZ) (Braunschweig, Germany). Organic solvents purchased from Fisher Scientific (Leics, UK).

## 3. Formulation of Bilosomes

Apigenin bilosomes (APG-BLs) were developed by a slightly modified version of the solvent-evaporation method [18]. The ingredients (CHL, PC, APG) were weighed and dissolved in chloroform-methanol mixture (1:1, 10 mL) for complete solubilization (Table 1). Separately, an aqueous surfactant/bile salt solution was prepared and heated at same temperature (40 °C). The organic solvent blend was added dropwise to surfactant solution with stirring. After complete evaporation of organic solvent (50 °C), sample was continuously stirred for 3 h to form bilayered vesicles. The prepared bilosomes (BLs) were collected and further sonicated using a bath sonicator to reduce their size [19]. The CH coating for APG-BLs was added via different chitosan concentrations (0.25%, 0.5%) in 0.5% *v*/*v* glacial acetic acid. CH solution was added dropwise to APG-BLs (F1-F6) using a syringe with continuous stirring at room temperature. The stirring was fixed at 100 rpm, and stirring was performed for 4 h [20]. Samples were collected and stored at room temperature for further characterization.

## 4. Vesicle Evaluation

The prepared apigenin bilosomes (APG-BLs) were evaluated via size analyzer (Malvern Zetasizer., Malvern, UK) to determine the different vesicles parameters. The prepared sample (0.1 mL) was taken and diluted with double distilled water. The dispersion was transferred to a cuvette to measure size and PDI. The ZP of diluted sample was evaluated in a separate cuvette. The ideal values of PDI and ZP for the nano-dispersion would be less than 0.5 and ± 30 mV to give a stable formulation. The chitosan-coated bilosomes were further evaluated for similar parameters to assess the difference in these characteristics.

## 5. Encapsulation Efficiency (EE)

EE of APG-BLs (F1-F6) and APG-CHBLs (F2C1-F2C2) was tested by indirect method. The samples (2 mL) were taken and centrifuged at 10,000 rpm in a cooling centrifuge (4 °C) for 3 h to separate free APG from supernatant [21]. The supernatant was collected and diluted to estimate the APG content at 338 nm using a UV spectrophotometer [1]. EE was calculated using Equation (1):

(1) $$\%\mathrm{EE}=\frac{\left(\mathrm{Total}\;\mathrm{APG}-\mathrm{Free}\;\mathrm{APG}\right)}{\mathrm{Total}\;\mathrm{APG}}\times 100$$

## 6. Transmission Electron Microscopy (TEM)

TEM study was performed to evaluate morphology of optimized chitosan-coated apigenin bilosomes (F2C1). The study was performed by taking one drop of sample on a grid and then adding uranyl acetate (2% *w*/*v*) as a contrast agent. The sample was kept aside for 5 min to complete staining process for visualization under a high resolution electron microscope (JOEL JEM1010, Japan).

## 7. In Vitro Drug Release

The release study was performed to evaluate release pattern from test samples (APG-BLs and APG-CHBLs) and compared with pure APG. Dialysis bag (MW 12,000 kDA) was used as membrane to check release behavior. The different formulations and pure APG dispersion (5 mg of APG) were transferred to the dialysis bag and both the ends of the membrane tied. The sample filled dialysis bag dipped into a beaker containing phosphate buffer saline, release media (500 mL with 20% ethanol *v*/*v*, pH 7.4). The release media was stirred with a magnetic stirrer (100 rpm), and temperature was fixed at 37 °C. The released content (5 mL) was removed at a fixed time and replaced with blank release media. The released samples were diluted and filtered, and the concentration of APG in each sample at each time point was measured using HPLC method [22].

## 8. Infrared Spectroscopy

The compatibility study between drug (APG), physical mixture and APG-CHBLs (F2C1) was evaluated using infrared spectroscopy. The sample of pure APG was compared with the spectra of the prepared formulation. The scanning was performed between 400 and 4000 cm^−1^. The changes in peak height and peak position in APG-CHBLs were evaluated to assess the interactions between pure APG and the carriers.

## 9. Mucoadhesive Study

The mucoadhesive study was performed via the adsorption method using mucin. The pure APG, APG-BLs (F2) and APG-CHBLs (F2C1) were taken and added to the standard mucin solution (1 mg/mL). The samples were added to the mucin standard solution (1:1) and then incubated for 3 h at room temperature. Each sample was centrifuged at 5000 rpm for 1 h and supernatant was collected. Mucin concentration in supernatant was estimated with an appropriate dilution. The amount of mucin was calculated with the equation below:

(2) $$\mathrm{Mucoadhesive}\;\mathrm{study}=\left(\frac{\mathrm{Ca}-\mathrm{Cb}}{\mathrm{Ca}}\right)\times 100$$

Ca: initial mucin concentration; Cb: free mucin concentration.

## 10. Permeation Study

APG permeation across the egg membrane for the prepared BLs (F2) and CHBLs (F2C1) was evaluated by the previously slightly modified method [23]. The study was performed to evaluate difference in the permeation from APG-CHBLs, APG-BLs and pure APG. The egg membrane was used to study the permeation because it is similar to intestinal membrane [24]. The diffusion cell having area of 3.22 cm^2^ with the receptor volume of 22 mL was used to assess the permeation behavior. The phosphate buffer saline with ethanol (20%) filled to the receiver cell and the temperature was fixed at 37 ± 0.5 °C. The released content was collected, and same volume of fresh media is replaced. The released content at each time point was filtered and evaluated for the amount of APG permeated across the membrane. The drug permeation (%) and flux (µg/cm^2^/h) from each sample were calculated. The flux value of pure APG was compared with formulations F2 and F2C1 to determine the enhancement in permeation flux.

## 11. Antimicrobial Study

The antimicrobial activities of the pure APG, APG-CH-BLs (F2C1) and standard drugs (imipenem, ceftazidime, nystatin) were evaluated with different microorganisms (*C. albicans*, *P. aeruginosa*, *E. coli* (50 µg/mL); *B. subtilis*, *S. aureus* (25 µg/mL)). The bacterial cultures were grown in nutrient broth, and each strain was grown to an optical density (OD) of 600 with a bacterial load equivalent of 5 × 10^6^ CFU/mL. The media were incubated for 24 h at 37 ± 0.5 °C, and then 1 mL of culture suspension was transferred and mixed thoroughly. The mixture was transferred to the sterilized Petri dish and kept aside for solidification of media. After solidification, a small well of 10 mm was made with a sterilized stainless steel borer. The sample (100 µL) was filled into each well, covered and kept aside. The petri-plates were inverted and incubated for 24 h to measure zone of inhibition of each well.

## 12. Cell Viability Study

The prepared APG-CHBLs (F2C1) were evaluated (via cell viability) for their efficacy against breast cancer (MCF7) and lung cancer (A549) cell lines. The findings of pure APG were compared with those of the APG-CHBLs (F2C1). MTT (3-(4,5-dimethylthiazol-2-yl)-2,5-diphenyl tetrazolium bromide) was used to assess effects of pure APG as well as APG-CHBLs. Cell lines (MCF7 and A549) were cultured in Dulbecco modified eagle medium (DMEM) (10% fetal bovine serum) with 15,000 cells per well. The cells were kept in an incubation chamber for 24 h to complete the growth of cells with a continuous flow of 5% CO_2_. The samples (pure APG and F2C1) were taken in different concentrations to evaluate cell viability. DMSO was used to prepare the stock solution and further diluted in a concentration range between 15.6 and 250 µM using cell culture media. The DMSO concentration was kept below 1% to avoid undesirable effects. The samples in triplicate were added to the wells and incubated for 24 h. After incubation, an MTT solution (10 µL, 5 mg/mL) prepared in phosphate buffered saline was added to each well. The treated samples were kept in an incubator for 4 h to metabolize MTT by viable cells. The media were replaced in each well, and DMSO was added to dissolve formazan solution to estimate the cell viability at 570 nm (DMSO was the control).

## 13. Statistical Analysis

The experiments were carried out in triplicate, and findings are presented as mean with standard deviation. One-way ANOVA has been used in the statistical analysis, followed by Tukey analysis between the different samples. Graph Pad Instat (Graph Pad software Inc., La Jolla, CA, USA) was used to conduct analysis.

## 14. Results and Discussion

APG-BLs were prepared by a solvent evaporation method using cholesterol (CHL), sodium cholate (SC) and phosphatidylcholine (PC). The prepared APG-BLs were further coated with the natural polymer chitosan to enhance the mucoadhesion. The formation of APG-CHBLs was mainly attributed to electrostatic interaction between positively charged CH and negatively charged BLs. The addition of positively charges to anionic BLs lead to changes in size and zeta potential. An increase in size may have been due to surface coating from CH and the formation of double-layered vesicles. The prepared APG-BLs had nano-metric VS, low PDI, negative ZP and high EE. Based on these findings, the selected formulation (F2) was further coated with CH (0.25 and 0.5%), and a marked increase in size and a positive zeta potential were observed (Table 2).

## 15. Vesicle Characterization

**Figure 2:**
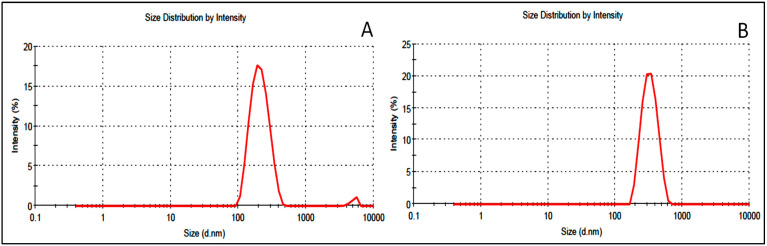
Vesicle size (**A**) apigenin bilosomes (F2) and (**B**) chitosan-coated apigenin bilosomes (F2C1).

The prepared APG-BLs and APG-CHBLs were evaluated for size, PDI and zeta potential (Table 2). A significant (*p* < 0.001) difference in the VS was found among APG-BLs as well as APG-CHBLs. APG-BLs (F1–F6) showed mean sizes of 211 ± 2.87 (F2) to 433 ± 1.98 nm (F3). The minimally-sized formulation F2 (211 ± 2.87 nm, Figure 2A) was further coated with two different concentrations of chitosan (0.25% *w*/*v*, 0.5% *w*/*v*), and the products were called APG-CHBLs (F2C1 and F2C2). We found a significant (*p* < 0.001) enhancement in size after coating with CH compared to formulation F2 (298 ± 3.56 nm vs. 354 ± 4.33 nm, Figure 2B). The chitosan concentration (0.25% or 0.5%) significantly (*p* < 0.001) affected the vesicle size. The literature reports that larger particles enter the lymphatic system, whereas particles under 500 nm in diameter use the endocytosis pathway for the transport of drugs [2]. In our study, APG-BLs and APG-CHBLs had particle sizes below 500 nm. This size can also enhance drug absorption due to availability of a greater surface area. PDI value did not show any significant variation in the results (0.13 to 0.39). APG-BLs and APG-CHBLs showed PDI values < 0.7 and considered as suitable delivery systems [25]. The surface charge on vesicle is very important for cellular interaction and uptake. A high negative zeta potential of the prepared APG-BLs indicates superior stability. The nano-sized APG-BLs showed values between −17 and −29 mV (±30 mV considered as stable) [26]. The values can be considered a distinguishing characteristic of APG-BLs. Flocculation exceeded repulsive forces [27]. The low ZP can be explained by the contribution of the lipids: the formation of negative charges in an aqueous environment [2]. The surface of each particle was completely covered with positively charged chitosan, and repulsion among the BLs thereby took place [28]. A positive charge of CH easily binds with negatively charged intestinal mucin and will help to increase drug properties [29].

## 16. Encapsulation Efficiency (EE)

The prepared APG-BLs was assessed for amount of APG entrapped in the bilosomes (Table 2). A significant (*p* < 0.001) differences in the EE (69.5 ± 0.2 to 81.5 ± 1.29%) were observed due to difference in BLs composition. The sample (F3) showed the minimum encapsulation efficiency of 69.5 ± 0.2%, having the composition CH:PC (4%) and T80 (5% *w*/*v*). Formulation F2 prepared with SDC:T80 (5%) had the maximum encapsulation efficiency (81.5 ± 1.29%). EE improved with an enhancement in the concentration of bile salts/surfactant (individually or together). From the results, it was observed that T80 or SDC alone did not significantly increase the EE. The blend of SDC:T80 (1:1) significantly (*p* < 0.05) enhanced EE compared to T80 or SDC alone. At high concentration, it could help to form mixed micelle and contribute to enhanced solubility in the dispersion medium [30,31]. The formulation (F2) further coated with chitosan (0.25% *w*/*v* and 0.5% *w*/*v*). A non-significant difference in the EE was observed in the APG-CHBLs (F2C1, F2C2). There was a slight alteration in the EE was also found between F2C1 (88.1 ± 1.48%) and F2C2 (90.1 ± 1.71%). Chitosan formed surface coating over the lipid bilayer of liposomes and prevented the leakage of the drug [32]. The formulation F2C2 showed slightly high EE due to the high concentration of chitosan (0.5%) as a coating polymer. The encapsulation of the drug depends upon the concentration of lipid and the concentration of polymer. The hydrophobic drug can easily entrap in the lipid bilayer.

## 17. TEM Evaluation

**Figure 3:**
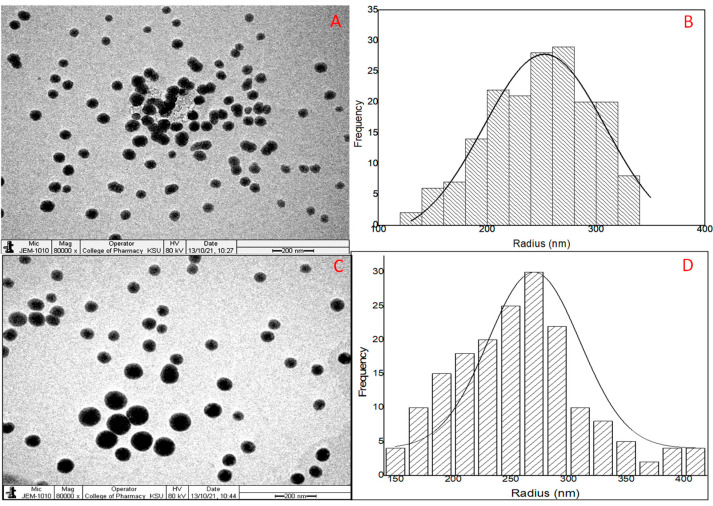
Apigenin bilosomes (F2): TEM image (**A**), Vesicle size distribution histogram (**B**); chitosan-coated apigenin bilosomes (F2C1): TEM image (**C**), Vesicle size distribution histogram (**D**).

The surface morphology of the prepared APG-BLs (F2) and APG-CHBLs (F2C1) showed non aggregated spherical structure (Figure 3A–C). The outer surface was found to be smooth and thin layer of coating was observed. The samples (F2, F2C1) also evaluated for size distribution curve and the image showed size distribution between 200 to 45 nm (Figure 3B–D). The distribution histograms are in close agreement with the results of TEM and DLS particle size image.

## 18. Drug Release (%)

**Figure 4:**
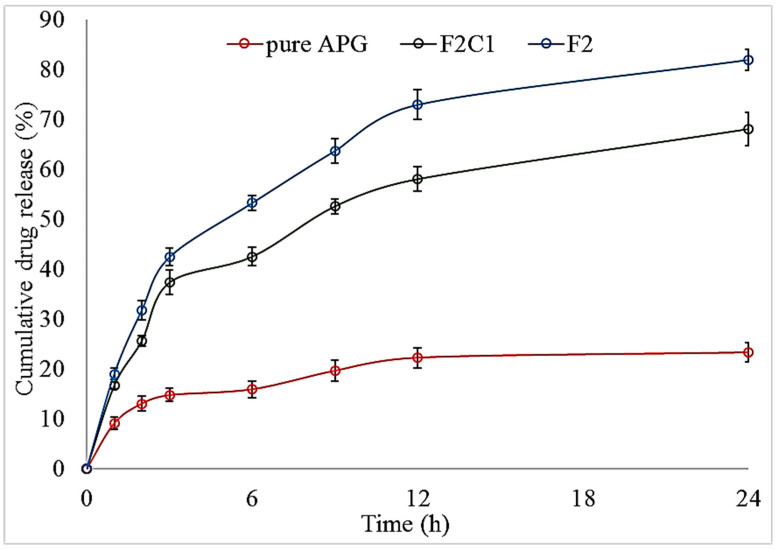
Release profiles of pure Apigenin (APG), apigenin bilosomes (F2) and chitosan-coated apigenin bilosomes (F2C1). The data are shown as means ± SDs (*n* = 3).

The drug release of pure APG, APG-BLs and APG-CHBLs were evaluated and the graph shown in Figure 4. The in vitro release data revealed higher APG release from APG-BLs (F2) and APG-CHBLs (F2C1). The data revealed that drug efflux from the tested formulations was found to be a biphasic mechanism. In initial 2 h, a quick release was achieved and later the prolonged-release was achieved. APG-BLs (F1-F6) showed a higher drug release than the pure APG in the tested time period (Table 2). The pure APG showed poor drug release (23.23 ± 0.94) from the dialysis bag due to poor water solubility. APG-BLs showed significantly higher release: 58.22 ± 0.73% (F3) to 81.9 ± 1.15% (F2) (*p* < 0.05). The higher drug release by APG was due to nano-metric vesicle size and availability of more effective surface area. An increase in the surface area leads to an increase in contact points of the drug in dissolution medium [4]. The presence of surfactant in BLs also helped to solubilize APG in dissolution media. Initially, a quick release occurred due to the availability of APG on the surfaces of the vesicles, and then slower release occurred. The slower drug release was due to encapsulated drug in inner core of BLs being released by diffusion and erosion or swelling of carrier [33]. For APG-CHBLs (F2C1, F2C2), the drug release was found to be slower than for APG-BLs. APG-CHBLs (F2C1) showed significantly slower release (62.86 ± 1.01 for 0.5% CH and 69.37 ± 1.74% for 0.25% CH; *p* < 0.05) during the last period of the experiment. The presence of an extra layer of chitosan helped to retard the release—which may be helpful for prolonging release in the body. The drug then needed to cross two layers to reach the release medium. The negative surfaces of the BLs were coated with chitosan via electrostatic interaction, reducing the release of APG [32].

## 19. Infrared (IR) Study

**Figure 5:**
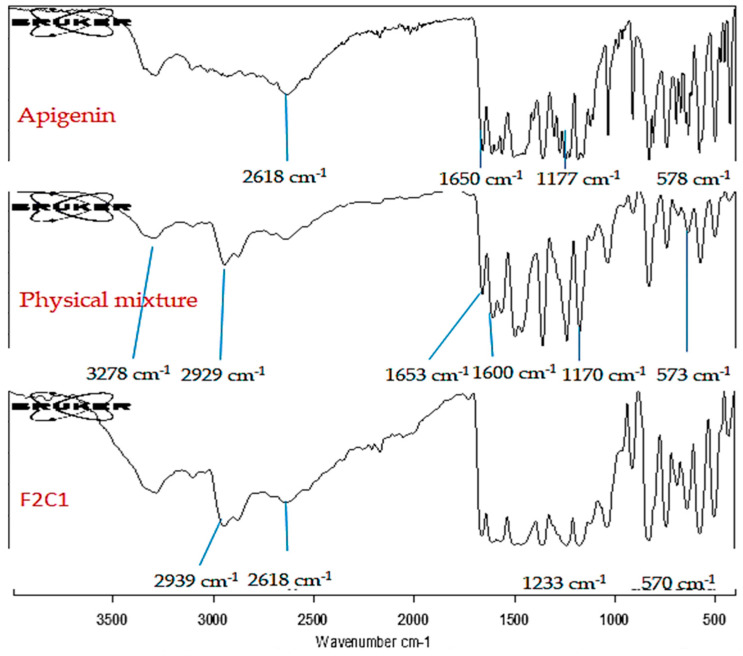
IR spectra of pure APG, physical mixture and chitosan-coated apigenin bilosomes (F2C1).

The IR assessment of APG, physical mixture as well as F2C1 were assessed. The spectra of all samples are portrayed in Figure 5. The pure APG depicted the characteristic peaks of stretching vibrations at 3276 cm^−1^ (OH group of phenol) and 2618 cm^−1^ (CH stretching); and 1650 and 1604 cm^−1^ (C=O) and 1177 cm^−1^ (C-O-C), functional group peaks. The physical mixture exhibited slight variations in the characteristic peaks of pure APG. It had peaks at 3278 cm^−1^ for the OH group of phenol; 1653 and 1600 cm^−1^ for C=O; and 1170 cm^−1^ for C-O-C. A C-H stretching peak at 2929 cm^−1^ and a stretching peak at 1235 cm^−1^ were observed for CH having a CH_2_-OH group. CHL and PC also showed a broad characteristic peak at 3327 cm^−1^ for OH group and a peak at 573 cm^−1^ signifying CN+ (CH_3_)_3_ deformation vibration. F2C1 showed no peak at 2618 cm^−1^ for CH stretching. This may have been due to solubility of APG in lipids. The CH_2_ symmetric stretching peak was observed (for CH) at 2939 cm^−1^. The carrier peaks were also found at 1233 cm^−1^ for carrier chitosan having a CH_2_-OH group. Another peek at 572 cm^−1^ was observed for CN+ (CH_3_)_3_. Slight changes in the characteristic peaks were observed for F2C1 compared to pure APG due to formation of vesicles.

## 20. Mucoadhesive Study (%)

The comparative mucoadhesive study of the APG-BLs (F2) and APG-CHBLs (F2C1) was performed while using mucin as an adsorbent. Mucin bound to the surfaces of the BLs and the concentration of mucin was calculated. The results are shown in Table 3. The presence of a greater concentration of mucin on the surfaces of a tested sample is depicted as a higher binding capacity. F2C1 showed a significantly higher binding capacity (68.77 ± 0.02%) than APG-BLs (23.6 ± 0.03%) (###, *p*< 0.001). The higher binding capacity was due to the cationic chitosan interacting with the anionic mucin, hydrophobic interactions and hydrogen bonding [34]. The cationic charge of chitosan confirms zeta potential result. APG-BLs (F2) showed the least mucoadhesion (2.8-fold) due to lesser binding of BLs to negatively charged mucin. A positively charged Ch-BLs could bind to negatively charged mucin secreted from the intestines, consequently improving the drug’s bioavailability and therapeutic activity [29]. Furthermore, the enhanced mucoadhesion will provide a longer residence time and help achieve enhanced permeation and therapeutic efficacy [35].

## 21. Permeation Study

This study was evaluated to determine amount of APG permeated across egg membrane. APG, with poor solubility, had poor drug permeation. The permeation profiles of pure APG, APG-BLs and APG-CHBLs are shown in Table 3. The pure APG showed poor permeability (31.11 ± 1.18 µg/cm^2^/h), significantly less than that of the APG-BLs (101.72 ± 4.06 µg/cm^2^/h) and APG-CHBLs (131.03 ± 7.57 µg/cm^2^/h) (***, *p* < 0.001). APG-BLs showing better permeation than the pure APG may have been due to the enhanced fluidity of the membrane in the former, possibly because of interaction of phospholipid molecules with membrane layer [36]. Diameter size till 200 nm is not a significant factor to the permeation of drugs [37]. The drug permeation level of APG-CHBLs (F2C1) was found to be significantly higher than those of pure APG and APG-BLs (F2). Among the three samples, the permeation profiles were as follows from high to low: APG-CHBLs > APF-BLs > pure APG. The chitosan-coated BLs showed enhanced permeation due to the presence of cationic charge on their surfaces. The positively charged surfaces of CH-BLs better interacted with negative charged membrane. The lesser permeation from APG-BLs is attributed to repulsion of formulation by negatively charged BLs, leading to reduced steady-state flux [38].

## 22. Antimicrobial Activity

**Figure 6:**
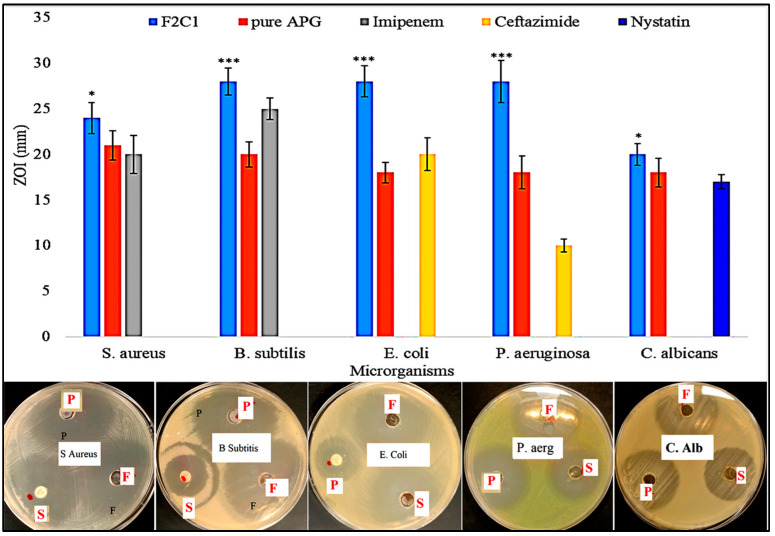
Antimicrobial profiles and zones of inhibition of pure apigenin, chitosan-coated apigenin bilosomes (F2C1) and standard compounds (imipenem, ceftazimide and nystatin). Results depicted as means ± SDs (*n* = 3). Tukey Kramer test was used to compare different groups. *** highly significant compared to pure APG, * significant compared to pure APG.

The prepared APG-CHBLs, pure APG and standards (imipenem, ceftazimide and nystatin) were evaluated and are compared in Figure 6. The pure APG showed the ZOI of 21 mm against *S. aureus*, 20 mm against *B. subtilis*, 18 mm against *E. coli*, 18 mm against *P. aeruginosa* and 18 mm against *C. albicans*. APG had a lesser effect on each organism due to poor solubility. APG is not able to permeate the cell walls of such organisms. APG encapsulated in CH-BLs showed significantly (*** *p* < 0.001, * *p* < 0.05) enhanced antibacterial activity. They had ZOI of 24 mm against *S. aureus*, 28 mm against *B. subtilis*, 28 mm against *E. coli*, 28 mm against *P aeruginosa* and 20 mm against *C. albicans*. The differences in the activity were found to be highly significant (*p* < 0.05) regarding *B. subtilis*, *E. coli* and *P. aeruginosa* compared to *S. aureus* and *C. albicans*. It showed activity against both Gram-negative and Gram-positive organisms. The difference in the activity against them was due to the permeability barrier provided by multilayered cell walls in Gram-negative bacteria; and they can break down foreign particles at the cell wall [39,40]. APG-CHBLs showed enhanced antibacterial activity compared to pure APG due to the nano-sized vesicles and enhanced solubility. The presence of bile salts and surfactant in the formulation led to higher solubility, which made a greater amount of APG available, enhancing therapeutic efficacy. In addition, the presence of chitosan on the surfaces of the APG-BLs has been reported to have antibacterial activity [41]. Chitosan showed the activity via binding with negative charged bacterial cell wall. It also helps to alter membrane permeability and inhibits DNA replication, leading to cell death [42]. The antibacterial activity was also tested for the standard compounds imipenem, ceftazimide and nystatin. Imipenem showed antibacterial activity against *S. Aureus* (20 mm) and *B. Subtilis* (25 mm). Ceftazidime depicted ZOI of 20 mm against *E coli* and 10 mm against *P. aeriginosa*; and Nystatin had a ZOI of 17 mm against *C. albicans.* The results found that the standard drugs showed slightly higher antibacterial activity than pure APG and lesser activity than APG-CHBLs. The combination of chitosan and APG showed synergistic action against the tested organisms, and made the vesicles ideal delivery systems.

## 23. Cell Viability

**Figure 7:**
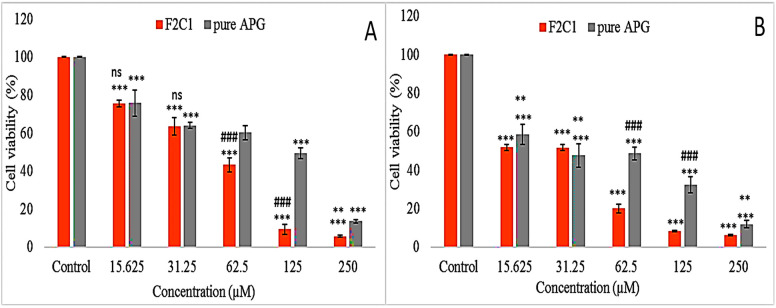
Cell viability studies of pure apigenin and chitosan-coated apigenin bilosomes (F2C1) against (**A**) lung cancer cell line A549 and (**B**) breast cancer cell line MCF7. Results depicted as mean ± SD (*n* = 3). Tukey Kramer test was used to compare different groups. *** highly significant compared to control, ** significant compared to pure APG, ### highly significant compared to pure APG, ns—non-significant.

The study of the prepared APG-CHBLs (F2C1) and pure APG used lung cancer cell line A549 (Figure 7A) and breast cancer cell line MCF7 (Figure 7B). The study showed a highly significant (*p* < 0.001) effect against each cell line in the concentration range of 15.6–250 µM compared to the control. A significant difference between the pure APG and F2C1 was also observed at the tested concentrations. The pure APG showed cell viability as follows against the A549 cell line: 250 µM (13.64%), 125 µM (49.49%), 62.5 µM (60.28%), 31.25 µM (64.1%) and 15.6 µM (75.83%) (Figure 7A). F2C1 showed greater activity at same concentrations: 250 µM (5.69%), 125 µM (9.43%), 62.5 µM (43.27%), 31.25 µM (63.6%) and 15.6 µM (75.68%). A significant difference in cell viability was observed at each concentration compared to control (*p <* 0.001). The effect was found to be concentration dependent. The maximum activity was observed at 250 µM from both samples. F2C1 depicted a 2.39-fold, significantly higher activity level than pure APG (*p* < 0.001). F2C1 showed significantly greater activity at 250, 125 and 62.5 µM, but there were non-significant differences in the activity at 31.25 and 15.62 µM. The IC50 values were also calculated for F2C1 and pure APG, and the difference was found to be significant (*p* < 0.001). The pure APG had an IC_50_ value of 107.61 µM and F2C1 had a 1.94-fold lower IC50 value (55.25 µM). APG showed activity on the lung cancer cell line, confirming its ability to induce apoptosis. Said apoptosis involves the mitochondrial pathway associated with APG–DNA interaction, DNA fragmentation, ROS accumulation and cytochrome c release [43]. In another study, APG induced apoptosis, cell death and depolymerized microtubules in A549 cells [44]. Cell viability studies with F2C1 and pure APG were also performed with a breast cancer cell line (MCF7). The results revealed significant (*p* < 0.001) differences (Figure 7B). The pure APG showed cell viability as follows: 250 µM (12.0%), 125 µM (32.28%), 62.5 µM (48.7%), 31.25 µM (47.6%) and 15.6 µM (58.6%)**.** F2C1 showed the greater activity at the same concentrations: 250 µM (6.3%), 125 µM (8.29%), 62.5 µM (20.1%), 31.25 µM (51.67%) and 15.6 µM (51.8%). A non-significant difference in cell viability was found for 15.62 and 31.25 µM treatments. Significant differences (*p* < 0.001) were observed for 62.5, 125 and 250 µM. The activity was found to be concentration dependent. As the concentration of APG increased, the cell viability decreased. F2C1 showed about 2 to 4-fold higher (*p* < 0.001) activity at 250 and 125 µM than pure APG. The IC_50_ values were calculated for pure APG and F2C1. The pure APG had an IC_50_ value of 42.86 µM, and F2C1 had a 2.26-fold lower IC_50_ value (18.93 µM). The difference was found to be highly significant (*p* < 0.001). It showed the activity against breast tumors in a dose-dependent manner [45]. It acts by suppressing proliferation and clonogenic survival of breast cancer. The death of cells takes place due to apoptosis caused by an elevated level of caspase 3 and a high Bax/Bcl-2 ratio [44,46]. From this study, it can be concluded that F2C1 showed significantly (*p* < 0.001) greater activity than pure APG due to enhanced solubility of APG in presence of surfactant. Due to its enhanced solubility, a higher concentration of APG reaches to target site. Chitosan coating also promotes anti-cancer activity of prepared formulation. It promotes drug permeation by enhancing the mucoadhesion and opening the tight junctions of cell walls, leading to accumulation of drug [47]. The low-molecular-weight CH possesses a cationic charge in its amino group, causing attraction towards cancer cells as a result of a higher negative charge of cancer cell than normal cells [48,49].

## 24. Conclusions

APG-BLs were prepared by using CH, PC and SDC in different ratios. The prepared formulations showed nano-metric vesicle size, negative zeta potentials, low PDI and high EE. Then, the selected formulation (F2) was coated with chitosan (0.25, 0.5% *w*/*v*) to enhance its mucoadhesion. The chitosan-coated APG-BLs (F2C1 and F2C2) were slightly larger, had a positive zeta potential, showed higher encapsulation efficiency as well as provided slower drug release. The chitosan-coated bilosomes (F2C1) showed significantly (*p* < 0.05) enhanced APG permeation and mucoadhesion. An antimicrobial and cell viability results revealed better activity than the pure APG. The cell viability assay results revealed concentration dependent activity against the tested lung cancer and breast cancer cell lines.

## Figures and Tables

**Table 1:** Formulation design of apigenin- bilosomes and chitosan coated apigenin bilosomes.

Formulations	CHL: PC (% *w*/*v*)	SDC: Tween 80 (% *w*/*v*)	Tween 80 (% *w*/*v*)	SDC (% *w*/*v*)	Chitosan (% *w*/*v*)
F1	4	5	-	-	-
F2	4	10	-	-	-
F3	4	-	5	-	-
F4	4	-	10	-	-
F5	4	-	-	5	-
F6	4	-	-	10	-
F2C1	4	10	-	-	0.25
F2C2	4	10	-	-	0.5

Sodium Deoxy Cholate (SDC); Phosphatidyl choline (PC); Cholesterol (CHL).

**Table 2:** Physicochemical data of apigenin bilosomes (APG-BLs) and chitosan-coated apigenin bilosomes (APG-CHBLs).

Formulation	Size (nm)	PDI	Zeta Potential (mV)	Encapsulation Efficiency (%)	Drug Release (%)
F1	361 ± 2.1	0.25	−23	73.1 ± 1.4	72.9 ± 0.9
F2	211 ± 2.9	0.35	−29	81.5 ± 1.3	81.9 ± 1.1
F3	433 ± 1.9	0.13	−15	68.9 ± 0.9	58.2 ± 0.7
F4	385 ± 3.6	0.21	−17	69.5 ± 0.2	64.9 ± 1.2
F5	334 ± 2.7	0.26	−20	73.7 ± 0.8	63.1 ± 1.6
F6	368 ± 3.1	0.29	−21	79.1 ± 1.6	66.2 ± 1.2
F2C1	298 ± 3.6	0.39	+17	88.1 ± 1.5	69.4 ± 1.3
F2C2	354 ± 4.3	0.43	+21	90.1 ± 1.7	62.9 ± 1.1
Pure APG					23.2 ± 0.9

**Table 3:** Permeation profiles and mucoadhesion results of apigenin-loaded bilosomes (F2) and chitosan-coated apigenin-loaded bilosomes (F2C1). Results depicted as means ± SDs (*n* = 3). Tukey Kramer test was used to compare different groups. *** highly significant compared to pure APG, ### significant compared to F2.

Parameters	Pure APG	F2	F2C1
Permeation (%)	9.77	31.94	41.14
Flux (µg/cm^2^/h)	31.11 ± 1.18	101.72 ± 4.06 ***	131.03 ± 7.57 ***
Mucoadhesive property (%)	-	23.6 ± 0.03	68.77 ± 0.02 ###

## Data Availability

The data presented in this study are available on request from the corresponding author.
